# Artificial intelligence in rheumatoid arthritis: potential applications and future implications

**DOI:** 10.3389/fmed.2023.1280312

**Published:** 2023-11-16

**Authors:** Vinit J. Gilvaz, Anthony M. Reginato

**Affiliations:** ^1^Division of Rheumatology, Department of Medicine, Rhode Island Hospital, Warren Alpert Medical School of Brown University, Providence, RI, United States; ^2^Department of Dermatology, Rhode Island Hospital, Warren Alpert Medical School of Brown University, Providence, RI, United States

**Keywords:** artificial intelligence, rheumatoid arthritis, machine learning, deep learning, clinical applications

## Abstract

The widespread adoption of digital health records, coupled with the rise of advanced diagnostic testing, has resulted in an explosion of patient data, comparable in scope to genomic datasets. This vast information repository offers significant potential for improving patient outcomes and decision-making, provided one can extract meaningful insights from it. This is where artificial intelligence (AI) tools like machine learning (ML) and deep learning come into play, helping us leverage these enormous datasets to predict outcomes and make informed decisions. AI models can be trained to analyze and interpret patient data, including physician notes, laboratory testing, and imaging, to aid in the management of patients with rheumatic diseases. As one of the most common autoimmune diseases, rheumatoid arthritis (RA) has attracted considerable attention, particularly concerning the evolution of diagnostic techniques and therapeutic interventions. Our aim is to underscore those areas where AI, according to recent research, demonstrates promising potential to enhance the management of patients with RA.

## Graphical abstract

Key concepts and definitions.

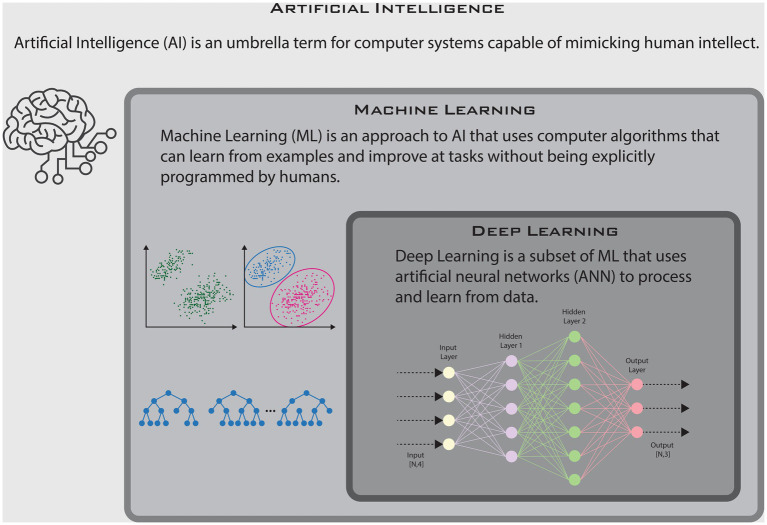

## Introduction

Over the past decade, AI has made a significant mark on healthcare, with applications spanning from radio diagnostics to drug discovery (1–3). Yet, its penetration in the field of rheumatology has been relatively slow when compared to other medical specialties (4). Today, the accelerated pace of innovation in AI, driven by big data, has made its impact on future healthcare too significant to ignore.

AI has the potential to fundamentally transform rheumatology by enhancing the diagnosis, treatment strategies, and overall management of rheumatic diseases.

AI is an overarching term that refers to general intelligent computing capable of mimicking human intellect. It encompasses both machine learning (ML) and deep learning. Broadly speaking, ML is an approach to AI that is designed to discern patterns from data, often autonomously and with minimal human supervision. What distinguishes ML from traditional statistical techniques is its capacity to learn from examples rather than being explicitly directed by predefined rules (5). Moreover, these models can adapt and improve in response to new information. Essentially, “learning” as they “experience” new data. ML models are initially trained on a labeled dataset (datasets where the outcomes are known), known as the ‘training set,’ and fine-tuned using a ‘validation set.’ The final model is then evaluated on an independent dataset, known as the ‘testing set,’ to obtain a final, unbiased measure of the model’s prediction capabilities or performance (sensitivity, specificity, accuracy, etc.) (6). Deep learning is a subset of ML that centers on algorithms based on artificial neural networks (ANNs)—i.e, multilayered abstract computational functions, loosely modeled after neuronal connections in the brain (7). Deep learning is a more potent tool, especially effective when working with complex data such as images, audio, and natural language. Most of the recent breakthroughs in AI have been powered by deep learning algorithms. These models perform best with very large datasets, making them ideal tools for analyzing and interpreting big data in healthcare. In this article, we have delved into a curated selection of AI-based research in RA published over the past 5 years. Our emphasis has been on studies that hold promise for practical clinical use, with the aim of describing their potential applications and future implications in the management of RA.

### Diagnosis and disease classification

A diagnosis of RA is usually based on a combination of clinical features, laboratory testing, and radiographic data (8). Today, ML and deep learning models trained on patient data are now capable of automating the process of identifying RA patients using similar parameters. A recent study by Bai et al. included a combination of patient demographic information and antibody profiles to accurately identify patients with RA using an artificial neural network (ANN) (9). Their model achieved an AUROC 0.95, and an F1 score (a metric that combines precision and recall) of 0.916, suggesting a high degree of accuracy. Of note, their model only utilized six features [age, sex, rheumatoid factor, anti-citrullinated peptide antibody (CCP), 14-3-3η, and anti-carbamylated protein (CarP) antibodies] to achieve this level of accuracy. Hand radiographs are also frequently used to make a diagnosis of RA in the appropriate clinical setting by way of pathogenomic abnormalities such as periarticular osteopenia and juxta articular erosions (10). To evaluate the utility of AI models in diagnosing RA using imaging, Üreten and colleagues developed a model to diagnose RA, using plain hand radiographs and convolutional neural networks or CNNs (a form of ANN used to identify and extract features from images) (11). The model was trained on a dataset containing radiographs from both RA patients and normal subjects. The final model achieved an accuracy of 73.33% with a low error rate of 0.0167 in identifying patients with RA. Their study demonstrates the potential of using CNNs to automate the diagnosis of RA based on a simple and inexpensive test like hand radiographs. Several other studies utilizing CNN have also achieved similar results in identifying radiographic features of RA using plain radiographs (12, 13). These and similar models could be used to assist primary care providers in the assessment of patients presenting with RA symptoms and prioritize specialty referrals.

The most recent classification criteria for RA was developed in part to identify patients with early disease as well as to identify a homogenous group of patients for enrollment in clinical trials (8). Although it serves as a valuable tool in identifying patients with RA, it stops short of sub-classifying patients. Recent research has focused on synovial tissue biopsies to help classify disease and guide therapy. In particular, synovial gene expression analysis has been used to develop RA subtypes based on the enrichment of specific inflammatory pathways (14). Orange et al. at the Hospital for Special Surgery in New York and in collaboration with the New York genome center, have taken this a step further. They applied machine learning to develop a synovial histological scoring system to predict gene expression subtypes (15). This was achieved in two steps. They first defined three distinct synovial gene expression subtypes, using k-means clustering (grouped as high, low, and mixed inflammatory subtypes). Histological features of the same synovial tissue were studied separately, and the 10 most common and reliable features were used for analysis. Using these predetermined histologic features as input and gene expression subtypes as labels, they trained a support vector machine (SVM) learning algorithm to predict the genomic subtype from histologic data alone. Models separating the high and low inflammatory subtypes performed best with an AUROC of 0.88 and 0.71, respectively. The validated histological scoring algorithm was even found to correspond to parameters of systemic inflammation (ESR, CRP) and antibody levels. Their study showcases the ability of distilling complex models to simple histological features that can be easily replicated at smaller centers. As the authors suggested, similar classification systems could also be useful in predicting poor response to anti-rheumatic drugs, especially in a patient where the mechanism of pain might not be attributable to inflammation.

Recent research into ‘multi-omics’ (genomics, transcriptomics, proteomics, and metabolomics) has provided a wealth of data that has also been used to develop AI models to identify RA patients with greater accuracy than traditional means (16–19). However, it will likely take more time before such advanced testing is made commercially available and for similar models to be replicated on a large scale.

### Detection of flares and disease activity monitoring

Flares are an important part of the disease process in patients with RA. They are typically defined as any worsening of disease activity that would, if persistent, lead to initiation or change of therapy (20). Flares can significantly impede physical activity and affect a patient’s quality of life. They are usually self-reported at scheduled visits or may even necessitate sick visits to help manage symptoms. Continuous disease monitoring to identify flares is not a common concept in inflammatory arthritis, unlike other diseases processes like diabetes (using continuous glucose monitors) or cardiac conduction abnormalities (utilizing wearable heart monitors) (21, 22). However, given that physical activity is often affected by flares, activity tracking has proven to be a good proxy for flare detection.

Using this rationale, Gossec et al. used data from activity trackers to detect flares in patients with RA (23). Using a consumer-grade wearable activity tracker, the physical activity of participants was monitored continuously over a 3-month period and patient-reported flares were collected using weekly questionnaires. It should however be noted that the flares were not assessed by a healthcare professional and only self-reported using by participants. They were nonetheless able to establish a clear relationship between the patient’s reported flares and a decrease in physical activity. This data was then used to help develop a ML model to help automate flare detection. In total, minute by minute tracking of physical activity provided nearly 13.5 million activity points. To analyze the large amount of data collected, a (multiclass) naïve Bayesian classifier was used. Of the total weekly data sets in the study, 70% (936 weeks) were used for training and the remaining 30% (403 weeks) were used as validation sets. Their model accurately detected both flares and absence of flare with a mean sensitivity of 95.7% and mean specificity of 96.7%. Given the popularity of these wearable devices, similar techniques could be used to continuously monitor RA disease activity, especially early in the treatment course. Patients with high flare rates could be prioritized for earlier clinic visits to consider therapy augmentation.

Today, point of care ultrasound (POCUS) has become a frequently used modality to both diagnose and monitor disease activity in RA (24). Images (including ultrasounds, radiographs, CT, and MRIs) are generally considered a great source of data for AI models, given the large number of data points (i.e., pixels) that are available for training. Access to large amounts of digital imaging data has enabled the development of deep learning models for image recognition and analysis. Deep learning approaches, specifically CNNs, have now become the gold standard in computer vision (25). They have been applied to doppler ultrasound images of patients with RA to detect diseased synovium and score disease activity (26). To achieve this, Anderson et al. developed a neural network using data from over thirteen hundred doppler ultrasound images. The images were labeled based on the 4-point OMERACT-EULAR Synovitis Scoring (OESS) system and scored from 0–3, where 0–1 was healthy and 2–3 indicated disease. The neural networks were then tested on a different data set of 176 images. For assessing healthy/diseased score, the neural networks highest accuracy compared with an expert rheumatologist were 86.4 and 86.9% with a sensitivity of 0.864 and 0.875 and specificity of 0.864 and 0.864, respectively. They even developed a neural network to automatically score the doppler images using the 4 class OESS system, which attained an average per-class accuracy of 75.0%, along with a quadratically weighted kappa score of 0.84 (a measure of agreement between ratings). Beyond synovitis, POCUS has also been helpful in assessing cartilage damage, which is known to be a strong predictor of physical disability in patients with RA (27, 28). Fiorentino et al. used CNNs to accurately identify the cartilage interface (margins) within the metacarpal joints and to make accurate thickness measurements. Their model proved to be very accurate, with a mean absolute difference (*ADF*) comparable to the intra-observer variability of skilled clinicians in the study (29). Similar models would go a long way toward automating POCUS measurements in other anatomical regions and aid in quantifying cartilage damage. The future of POCUS in rheumatology will definitely benefit from incorporation of similar deep learning models to enable real time image analysis and interpretations.

Irreversible joint damage is another marker of disease progression that can be monitored with periodic radiographs. Several scoring systems have been implemented to quantify radiographic changes in RA that typically use a combination of joint space narrowing and erosion to quantify joint damage (30). However, these scorings systems can be cumbersome to implement and affected by interobserver variability. To automate the this process, Hirano et al. developed a deep learning model to identify and assess joint damage on hand radiographs (31). They achieved this in two steps; they first used a ML model to detect the small finger joints (MCP, PIP etc.) and then used a deep learning model (CNN) to score joint destruction (utilizing the Sharp/van der Heijde method). Beyond CNNs, newer approaches utilize the faster and more efficient You Only Look Once (YOLO) model, has also proven to be accurate for both joint detection and for scoring of joint damage (12, 32, 33). These and similar techniques could provide an unbiased method for evaluating radiographic images and address issues like interobserver interpretation in both clinical practice and pharmaceutical trials.

### Choice of therapy and predicting outcomes

When it comes to the management of patients with RA, drug selection can be challenging, especially in patients that fail first-line therapy with methotrexate. Despite the popularity of anti-TNF therapies, multiple studies have shown that nearly 40% of patients respond poorly to these treatments (34). As the effectiveness of these therapies in individual patients are usually determined on a trial-and-error basis, multiple efforts have been made to identify better markers of drug response. Using a combination of demographic, clinical, and genetic markers, Guan et al. developed a Gaussian process regression (GPR) model that could predict changes in disease activity scores (DAS) and identify non-responders to anti-TNF treatment (35). The model was developed and cross-validated using data from 1,892 RA patients, which was then evaluated using an independent dataset of 680 patients. All patients had at least moderate disease activity at baseline with a DAS score > 3.2. The model predicted changes in DAS scores 24 months from baseline with a correlation coefficient of 0.406 and correctly classified the responses of 78% of subjects with an AUROC of 0.66. However, the data points used in their model, like patient genetic information, are not freely available and would limit the widespread implementation of similar models. Moreover, as GPR models are not parametric models, interpretability can become an issue. When making treatment decisions based on complex ML models, interpretability is key in minimizing bias and ensuring transparency. A set of approaches termed XAI, or explainable artificial intelligence, is increasingly utilized in healthcare research to help us understand the rationale behind the output of a ML algorithm (36). Using this approach, Koo et al. developed a ML model to predict the likelihood of achieving remissions in RA patients treated with biologic disease-modifying anti-rheumatic drugs (bDMARDs) at 1 year follow-up (37). The model analyzed registry data from 1,204 RA patients to identify key clinical features (age, duration of disease, inflammatory marker levels, antibody profile etc.) that would predict response to a variety of biological agents (adalimumab, etanercept, infliximab, golimumab, abatacept, and tocilizumab). Similar interpretable models could go a long way in ensuring optimal drug selection and avoid unwanted expenses and side effects in non-responders (Table 1).

**Table 1:** Selected reports of machine learning and deep learning algorithms in rheumatoid arthritis.

Task	Performance	Publication
Automate diagnosis of RA using patient demographics and antibody profile	AUROC: 0.951	Bai et al. (9)
Automate identification of RA from hand radiographs	Sensitivity: 0.6818 Specificity: 0.7826 Accuracy: 0.7333 FI score: 0.7143	Üreten et al. (11)
Predicting synovial gene expression subtypes (high^a^ and low^b^ inflammatory groups) from histological data	^a^ AUROC: 0.88 ^b^ AUROC: 0.71	Orange et al. (15)
Automated RA flare detection using activity trackers	Mean sensitivity: 95.7% Mean specificity: 96.7%	Gossec et al. (23)
Detecting synovitis from doppler ultrasound images	AUROC: 0.93.	Anderson et al. (26)
Predicting response to anti-TNF therapy	AUROC: 0.66	Guan et al. (35)
Predicting response to different biologic treatments	AUROC range for different therapies: 0.511–0.694	Koo et al. (37)
Predicting future disease activity using EHR data	AUROC 0.91	Norgeot et al. (38)
Predicting difficult to treat RA (D2T RA)	AUROC 0.73	Messelink et al. (39)

The widespread implementation of electronic health records (EHR) has resulted in the accumulation of large amounts of data for each individual patient. Numerous studies have highlighted the ability of ML and deep learning techniques to use EHR data to predict clinical outcomes (40–42). Similar success was seen by researchers at the University of California, who used structured EHR data to predict RA disease activity at their next clinical visit (38). The data used for analysis included demographic information, laboratory data, medication lists, and prior disease activity-measured using the clinical disease activity index (CDAI) score. Data was collected from 578 patients at a university hospital and 242 patients from a public safety net hospital. Their model achieved an AUROC of 0.91 in a test cohort of 116 patients at the university hospital and an AUROC of 0.74 in a test cohort of 117 patients at the safety net hospital. It should be noted that significantly different patient populations and treatment patterns were seen at each facility. Their study nonetheless highlights the ability of deep learning to build accurate prognostication models using EHR data alone.

As previously discussed, a significant percentage of RA patients still fail to respond to multiple therapeutic trials. Patients who do not respond adequately even after using two or more biological DMARDs or targeted synthetic DMARDs (tsDMARDs) with different mechanisms of action are recently termed to have difficult-to-treat RA (D2T RA) (43). To identify patients with D2T RA, Messelink et al. employed several approaches, including text mining and feature weighting, on a real-world database of RA patients (39). They further developed a model to predict the risk of developing D2T RA using data prior to prescribing the first biological or tsDMARD. Their model correctly predicted 79% of D2T RA patients, achieving an AUROC of 0.73. Their study highlights the potential of ML models in predicting treatment trajectories and the utility of unstructured real-world patient data in developing these models. Although D2T RA can result from various factors from poor adherence to comorbid illness, stratification of patients early in the disease course will certainly aid in closely monitoring high-risk patients.

### Looking to the future

Despite all the progress made in the field of AI, widespread implementation in healthcare has been relatively slow due to several factors. A primary obstacle is the limited access to healthcare data for training and testing models, which is largely due to the confidential nature of patient records (44). Additionally, healthcare data often exists in an unstructured and fragmented format, tailored more for human use rather than data analysis. When it comes to big data, health care is one of the fastest growing segments, growing nearly 50% year on year (45). Within the realm of rheumatology, the European League Against Rheumatism (EULAR) pioneered the development of consensus guidelines for handling big data, addressing its ethical dimensions, potential advantages, and the overarching goal of using big data to improve patient care (46). The American College of Rheumatologists (ACR) has also developed its own national EHR-enabled rheumatology registry as a source of big data (47). AI-driven approaches would serve as a valuable tool in leveraging these data sets to further research in rheumatology.

Progress in AI has come from both academia and industry. However, in recent years, industry has taken the lead in developing and commercializing AI-powered products and services (48, 49). The recent involvement of large technology companies in healthcare has catalyzed existing trends and moved us closer to commercially viable applications. Recent advancements have prominently featured “Foundation Models” —i.e., models trained on massive amounts of unlabeled data that excel in diverse tasks (50). Large Language Models (LLMs) are prime examples of these foundation models, adept at ‘understanding’ and generating natural language. They can provide detailed answers to intricate questions and facilitate more natural interactions with computers (51). Efforts are now being made to develop LLMs that can be combined with other AI tools to assist with medical decision making.

Recent iteration of these models has shown promise in answering USMLE (United States Medical Licensing Examination) style questions with great accuracy (52) and are being tested for they ability to serve as clinical decision support systems (CDSS). Efforts are also being made to embed AI models within the EMR to improve patient care in real time (53).

Despite all the progress made, it is important to remember that we are still in the early stages of AI-powered decision making. AI as we know it today is still essentially pattern recognition, masquerading as intelligence. Almost all the current AI-based research in healthcare has been done using retrospective data to both train and validate models (54). The quality and reliability of these models are also heavily dependent on the quality of data used to develop them. Inaccurate or non-representative data could easily lead the algorithm to arrive at erroneous predictions.

Interpretability is also another concern. Most of the complex deep learning algorithms today, although good at making predictions, offer little to no explanation as to how they arrived at those conclusions, essentially serving as a “black box” (55). The more complex the model, the less interpretable they become. This is definitely a cause of concern in the healthcare sector, where accuracy and reliability are of utmost importance. Moreover, LLMs, like the one described above, come with their own set of concerns. Despite their ability to improve interactions with humans using conversive language, these models have been known to “confabulate” or “hallucinate” responses when posed with questions outside their capabilities (51, 56). Although efforts are being made to overcome some of these challenges (36, 57), it is important to temper our expectations with the advances being made.

Like other aspects of healthcare, AI tools will need to be subject to high levels of regulation before we see widespread adoption. Several efforts have been made in this direction by both American and European regulatory agencies. The FDA recently issued the ‘AI/ML-Based Software as a Medical Device (SaMD) Action Plan’, which supports the development of methodologies for the evaluation and improvement of AI algorithms (58). However, currently, only the European Union has enacted actionable regulation through the European Medical Device Regulation (EU-MDR) that aims to enhance the scrutiny of AI tools in healthcare (59). We are likely to see more work in this space as medical devices and software become increasingly reliant on AI.

As we move toward a future powered by both humans and computers, it is important to ensure active physician participation when developing models that would influence patient care. To this end, efforts should also be made to incorporate elements of AI in medical education in order to inform future physicians about technology that will be used to care for patients. While AI offers immense potential in rheumatology and broader medical fields, its integration must be approached with care and responsibility.

## Conclusion

Ongoing advancements in AI technologies and their successful implementation in pilot studies are promising indicators of the future of RA patient care. Although significant progress has been over the past decade, several technical and regulatory obstacles need to be overcome before AI can be implemented in routine clinical practice.

## Author contributions

VG: Writing – original draft, Writing – review & editing. AR: Conceptualization, Supervision, Writing – original draft, Writing – review & editing.
